# Supplementary material for: Genome Based Meta-QTL Analysis of Grain Weight in Tetraploid Wheat Identifies Rare Alleles of GRF4 Associated with Larger Grains
Source: Genes (Basel). 2018 Dec 17;9(12):636. doi: 10.3390/genes9120636 (PMC6315823; doi:10.3390/genes9120636)
Supplement: Supplementary file 2 [file genes-09-00636-s002.pdf]

Genome based meta-QTL analysis of grain weight in tetraploid wheat identifies rare alleles of *GRF4* associated with larger grains

Raz Avni[1], Leah Oren[1,2], Gai Shabtai[1], Siwar Assili[2,5], Curtis Pozniak[3], Iago Hale[4], Roi Ben-David[5], Zvi Peleg[2], Assaf Distelfeld[1*]

Supplementary Materials

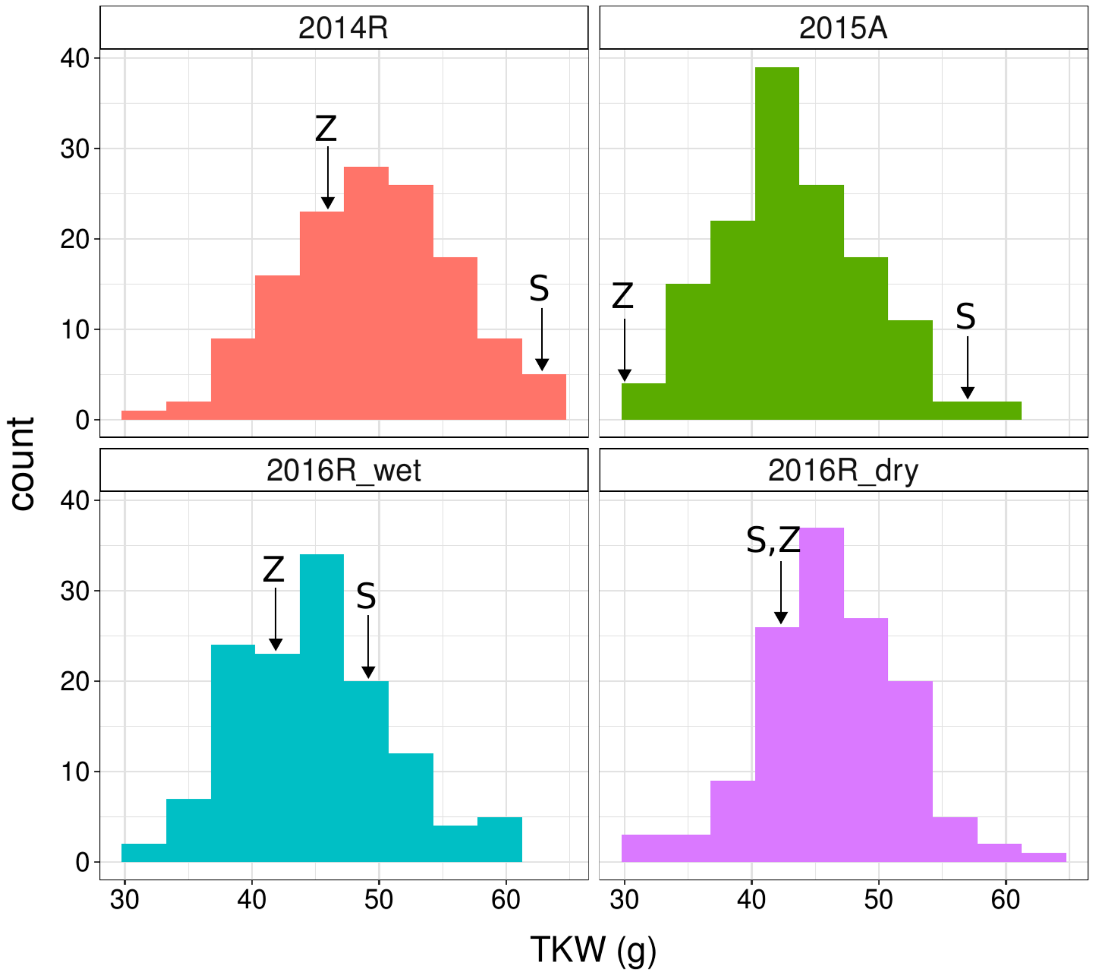

Figure S1. Distributions of thousand-kernel weight (TKW) on the Svevo × Zavitan population across four environments (2014R, 2015A, 2016R_wet and 2016R_dry). Arrows point to the mean value for the two parents Svevo (S) and Zavitan (Z).

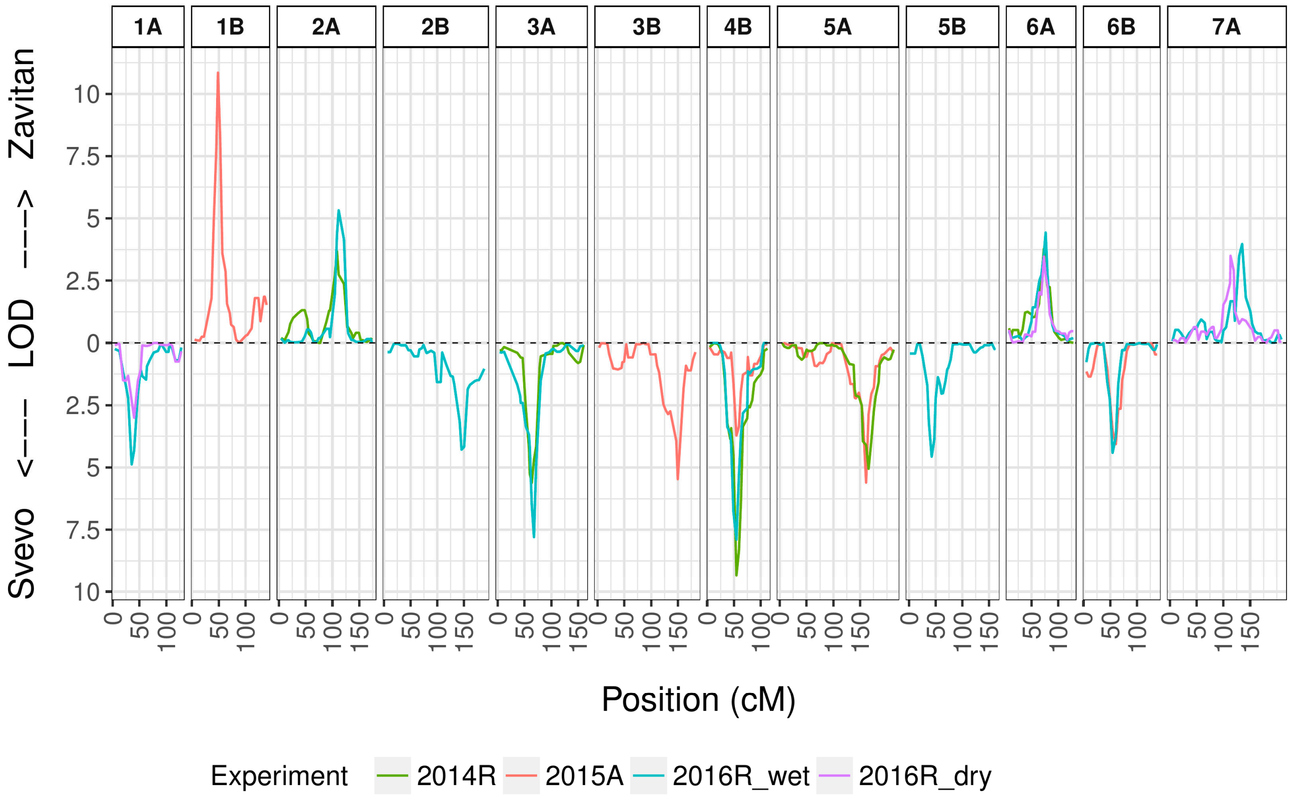

Figure S2. QTL analysis for TKW across four experiments. The x-axis shows the genetic position (cM) and the y-axis shows the LOD score, with the source of the high-TKW allele indicated (Zv = above x-axis; Sv = below x-axis).

Table S1. QTL parameters for TKW in four environments (see Table 1.)

Chromosome	Environment	LOD	P-value	PEV[a]	Subst.effect[b]	Genetic Marker	Start on Zavitan	End on Zavitan
1A	2015A	3.006	0.00093	0.059	2.731	IWA2056	31274191	31274391
1A	2016R_ WET	4.877	0.01429	0.069	2.76	IWA8551	23748366	23748266
1B	2015A	10.85	0.01429	0.209	-5.147	IWB20542	373810959	373810859
2A	2014R	3.682	0.00093	0.068	-2.949	IWB50818	608897808	608897908
2A	2016R_ WET	5.322	0.02703	0.051	-3.09	IWB2683/IWB44472	643855130	643855230
2B	2016R_ WET	4.284	0.00187	0.057	2.684	IWB46299	729462816	729462877
3A	2014R	5.622	0.00093	0.111	3.757	IWB53527/IWB52086	416199186	416199259
3A	2016R_ WET	7.795	0.01429	0.101	4.363	IWB16112	492095032	492094831
3B	2015A	5.471	0.01429	0.085	3.291	IWB7540	787686011	787686111
4B	2014R	9.331	0.01429	0.06	2.756	IWB72369/IWB72367	494965971	494966071
4B	2015A	3.719	0.00093	0.131	4.081	IWB72369/IWB72367	494965971	494966071
4B	2016R_ WET	7.896	0.01429	0.179	5.799	IWB72369/IWB72367	494965971	494966071
5A	2014R	5.052	0.01429	0.093	4.183	IWB42031/IWB25205	622359795	622359895
5A	2015A	5.608	0.01429	0.095	3.462	IWB686	611738001	611738101
5B	2016R_ WET	4.565	0.0028	0.056	2.678	IWB33375	383073895	383073979
6A	2014R	3.784	0.01429	0.083	-3.033	IWB31050	480981917	500982017
6A	2016R_DRY	3.47	0.00093	0.053	-2.602	IWB31050	520981917	500982017
6A	2016R_ WET	4.431	0.01429	0.055	-3.225	IWB31050	531470572	531470672
6B	2015A	4.063	0.01429	0.061	2.77	IWB72854	130241883	130241983
6B	2016R_ WET	4.411	0.00093	0.053	2.606	IWB19912	95059978	95060046
7A	2016R_DRY	3.5	0.01429	0.082	-3.028	IWA7741	579644500	579644700
7A	2016R_ WET	3.97	0.00093	0.049	-2.492	IWA6562	641817495	641817695

[a] Proportion of explained variance of the trait.

[b] The adaptive effect of an allele calculated as one-half of the mean difference between homozygotes with and without the allele.

Table S2. Significance of 6A TKW QTL between Svevo and Zavitan alleles in the RIL population. RILs were grouped by their parental allele at the peak QTL marker – IWB31050, significance was determined using a t-test. The table shows mean ± standard error.

	Svevo Allele	Zavitan Allele	pv
2016R-WL	45.5±0.7	47.4±0.6	0.035
2016R- WET	43.8±0.7	46.3±0.8	0.014
2014R	48.1±0.8	51.4±0.9	0.005

Table S3. Mean TKW of parental lines and segregating populations in the 9 independent studies used for the meta-QTL analysis.

Study	Population	Mean TKW (g) of the wild/emmer parent	Mean TKW (g) of the durum/emmer parent	Mean TKW (g) of the population	Type of cross
Elouafi and Nachit 2004	BC_1F_8 RIL	28.6	32.1	29.9	DW × WEW
Peleg et al. 2011	RIL	42	46	42	DW × WEW
Peng et al. 2003	F_3	10	30	-	DW × WEW
Thanh et al. 2013	F_2	19	52 (emmer)	-	DEW × WEW
Faris et al. 2014	RIL	28.84 (emmer)	55.12	40.84	DW × DEW
Russo et al. 2014	RIL	58, 45.5 (emmer)	74, 54.5	58.9, 56.9	DW × DEW
Golan et al. 2015	RIL	42	49.7	-	DW × WEW
Tzarfati et al 2014	RIL	48, 45	56, 51	-	DW × WEW
Avni et al. 2018	RIL	45.8, 29.7	61.8, 56.5	49.6, 43.1	DW × WEW

Table S4. Genotypes used for allelic diversity study with molecular marker for the presence of *GRF4-Az* and *GRF4-Ag*

Label	Location	Accession	Species	Improvement status	*GRF4*_allele
WE-1	Central Israel	PI 471021	dicoccoides	wild	-
WE-2	Northern Israel	PI 538673	dicoccoides	wild	-
WE-4	Central Israel	PI 471038	dicoccoides	wild	-
WE-6	Qazerin, Syria	PI 466946	dicoccoides	wild	-
WE-7	Northern Israel	Qazerin (UH 5)	dicoccoides	wild	-
WE-8	Northern Israel	PI 466957	dicoccoides	wild	-
WE-9	Northern Israel	Nesher (UH 27)	dicoccoides	wild	-
WE-10	Central Israel	PI 471060	dicoccoides	wild	*GRF4-Az*
**WE-11**	**Northern Israel**	**Zavitan**	**dicoccoides**	**wild**	*GRF4-Az*
WE-12	Northern, Israel	PI 467008	dicoccoides	wild	*GRF4-Az*
WE-14	Central Israel	PI 470962	dicoccoides	wild	-
WE-15	Central Israel	PI 466950	dicoccoides	wild	-
WE-16	Central Lebanon	PI 428132	dicoccoides	wild	-
WE-17	Central Lebanon	PI 352322	dicoccoides	wild	-
WE-18	Northern Israel	Mt. Hermon (UH 1)	dicoccoides	wild	-
WE-19	Central Israel	Mt. Gerizim (UH 17)	dicoccoides	wild	-
WE-20	Halab, Syria	PI 487264	dicoccoides	wild	-
WE-21	Iraq	Iraq (UH 41)	dicoccoides	wild	-
WE-22	Central Lebanon	PI 428129	dicoccoides	wild	-
WE-23	Central Turkey	PI 428066	dicoccoides	wild	-
WE-24	Diyarbakir, Turkey	PI 428084	dicoccoides	wild	-
WE-25	Karacadag, Turkey	PI 538666	dicoccoides	wild	-
WE-26	Diyarbakir, Turkey	PI 428054	dicoccoides	wild	-
WE-29	Diyarbakir, Turkey	PI 538642	dicoccoides	wild	-
WE-30	Diyarbakir, Turkey	PI 428072	dicoccoides	wild	-
WE-31	Central Turkey	PI 428070	dicoccoides	wild	-
WE-32	Diyarbakir, Turkey	PI 428036	dicoccoides	wild	-
WE-33	Diyarbakir, Turkey	PI 428025	dicoccoides	wild	-
WE-34	Diyarbakir, Turkey	PI 538631	dicoccoides	wild	-
G18-16	Gitit, Israel		dicoccoides	wild	*GRF4-Ag*
DE-1	Oman	PI 532302	dicoccum	domesticated	-
DE-2	India	PI 322232	dicoccum	domesticated	-
DE-3	Central Turkey	PI 319868	dicoccum	domesticated	-
DE-4	Central Turkey	PI 319869	dicoccum	domesticated	-
DE-5	Central Israel	PI 352347	dicoccum	domesticated	-

DE-6	Southern Turkey	PI 355454	dicoccum	domesticated	-
DE-7	Central Israel	PI 355496	dicoccum	domesticated	-
DE-8	Central Israel	PI 352357	dicoccum	domesticated	-
DE-10	Central Israel	PI 352367	dicoccum	domesticated	-
DE-11	Southern Turkey	PI 352352	dicoccum	domesticated	-
DE-12	Central Turkey	PI 182743	dicoccum	domesticated	-
DE-14	Italy	PI 352361	dicoccum	domesticated	-
DE-15	Spain	PI 191091	dicoccum	domesticated	-
DE-16	Spain	PI 276007	dicoccum	domesticated	-
DE-17	Central Turkey	PI 606325	dicoccum	domesticated	-
DE-18	Central Turkey	PI 352329	dicoccum	domesticated	-
DE-19	Ukraine	PI 94741	dicoccum	domesticated	-
DE-20	Slovenia	PI 377658	dicoccum	domesticated	-
DE-21	Croatia	PI 264964	dicoccum	domesticated	-
DE-22	Bosnia and Herzegovnia	PI 434995	dicoccum	domesticated	-
DE-23	Iran	PI 254158	dicoccum	domesticated	-
DE-24	Iran	PI 254169	dicoccum	domesticated	-
DE-26	Central Turkey	PI 470739	dicoccum	domesticated	-
DE-27	Central Turkey	PI 470738	dicoccum	domesticated	-
DE-28	Armenia	PI 94661	dicoccum	domesticated	-
DE-29	Central Turkey	PI 470737	dicoccum	domesticated	-
DE-30	Georgia	PI 326312	dicoccum	domesticated	-
**DDW**	**Italy**	**Svevo**	**Durum**	**domesticated**	**-**
